# Evidence-based criteria for identifying at-risk individuals requiring liver disease screening

**DOI:** 10.1097/HC9.0000000000000679

**Published:** 2025-03-21

**Authors:** Fredrik Åberg, Ville Männistö, Juho Asteljoki, Veikko Salomaa, Antti Jula, Annamari Lundqvist, Satu Männistö, Markus Perola, Panu K. Luukkonen

**Affiliations:** 1 Transplantation and Liver Surgery, Helsinki University Hospital and University of Helsinki, Helsinki, Finland; 2 Department of Medicine, University of Eastern Finland, Kuopio, Finland; 3 Department of Medicine, Kuopio University Hospital, Kuopio, Finland; 4 Minerva Foundation Institute for Medical Research, Helsinki, Finland; 5 Department of Internal Medicine, University of Helsinki, Helsinki, Finland; 6 Abdominal Center, Helsinki University Hospital, Helsinki, Finland; 7 Finnish Institute for Health and Welfare, Helsinki, Finland

**Keywords:** chronic liver disease, cirrhosis, population health, risk factors, screening

## Abstract

**Background:**

Liver fibrosis screening is recommended in at-risk groups, but a clear definition of “at risk” for entry criteria is lacking. We analyzed different combinations of established risk factors to define specific screening entry criteria with a prespecified sensitivity requirement.

**Methods:**

Data regarding individuals aged 40–70 years from Finnish health-examination surveys (FINRISK 2002–2012 and Health 2000, n=15,057) and the UK Biobank (n=454,990) were linked with healthcare registries for liver cirrhosis-related events (LREs; liver-related hospitalizations, cancer, or death). The predictive performance of 1919 combinations of risk factors, including alcohol consumption, metabolic disturbances, abnormal liver function tests, and Chronic Liver Disease risk score, was assessed for 10-year LRE risk requiring a minimum 90% sensitivity. Validations were performed using liver stiffness measurement (LSM) >12 kPa in the NHANES 2017–2020 sample (n=3367).

**Results:**

Optimal entry criteria for predicting 10-year LRE risk with >90% sensitivity included any one of: hazardous alcohol use, severe obesity, metabolic syndrome, an AST-to-ALT ratio >0.8 with elevated ALT, and an intermediate-to-high Chronic Liver Disease risk score. The sensitivity and specificity for this strategy were 91% and 51% for LREs, respectively, in the Finnish cohort, and 91% and 41% for LSM >12 kPa in the US sample. In the US sample, applying these entry criteria followed by fibrosis-4 ≥1.3 for predicting LSM >12 kPa reduced the sensitivity to 45% (specificity: 85%), which was attributed to the suboptimal sensitivity of fibrosis-4.

**Conclusions:**

This study identifies an inexpensive risk factor-based strategy with >90% sensitivity for predicting LRE and LSM >12 kPa, which is practical and scalable for targeted liver fibrosis screening to improve population outcomes. However, a more sensitive first-line noninvasive fibrosis test is needed.

## Graphical abstract

**Figure:**
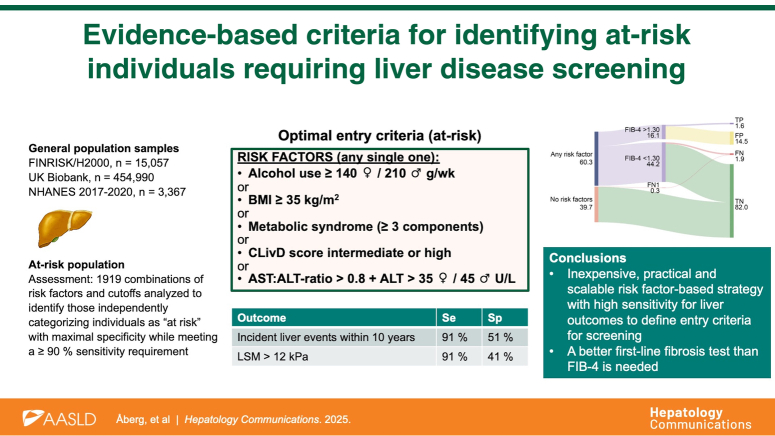

## INTRODUCTION

Screening of at-risk groups for advanced liver fibrosis using noninvasive fibrosis tests, including the Fibrosis-4 (FIB-4) Index and liver stiffness measurement (LSM), is essential for prompt detection of progressive disease.^1^^–^^4^ However, the precise definition of the target group based on risk factors to be used as entry criteria for fibrosis screening remains unclear. For population-level screening, it is essential to identify individuals with an increased risk of clinical liver outcomes rather than limiting the approach to those at the highest risk, to maximize the impact of downstream interventions on population-level morbidity and mortality. Guidelines discourage screening for steatosis or fibrosis in unselected populations.^1^^–^^4^

Harmful alcohol use, obesity, type 2 diabetes, and elevated liver function tests (LFTs) are considered the main risk factors for chronic liver disease. Community-based studies have also applied these risk factors as a trigger for fibrosis screening with FIB-4 or similar tests, improving case detection rates.^5^^–^^8^ These studies, however, could not determine the total number of cases with advanced liver fibrosis or liver-related events (LREs) identified or missed by this strategy, given the applied definitions for the target screening population. The optimal cutoff values for adiposity and alcohol use for the sake of liver disease screening, and the role (inclusion/exclusion) of other components of the metabolic syndrome, remain unclear. Furthermore, defining at-risk groups based on the presence/absence of single strong risk factors overlooks cases involving the coexistence of several weaker risk factors.^9^ Quantifying an individual’s overall risk by considering multiple risk components using prediction scores might better identify at-risk individuals.^10^

An improved evidence-based definition of at-risk individuals requiring liver fibrosis screening is crucial for effective screening programs that identify those at risk for clinical liver-related outcomes, not just liver fibrosis.^11^ This is because, on a population scale, there are generally low risks for clinical liver-related outcomes even with significant liver fibrosis present.^12^ Moreover, the risk-factor strategy should have a sufficiently high sensitivity to ensure the screening program can effectively reduce liver disease morbidity and mortality at the population level by capturing most patients and linking them to effective care. However, there are no established benchmark sensitivity levels for this purpose. We aimed to analyze different combinations of established risk factors to define entry criteria for liver fibrosis screening that maximize specificity while maintaining a minimum predetermined sensitivity. We focused on incident clinical LREs as the outcome in longitudinal settings, since this is the outcome that really matters, but we validated the findings also for elevated liver stiffness cross-sectionally.

## METHODS

### Finnish datasets used for longitudinal analyses

Data were sourced from the FINRISK studies conducted in 2002, 2007, and 2012, as well as the Health 2000 study. The FINRISK studies were standardized cross-sectional population-based surveys conducted every 5 years since 1972 by the National Public Health Institute (Finnish Institute for Health and Welfare since 2009) to assess risk factors for chronic diseases in adults aged 25–74 years drawn from the Finnish Population Information System. These studies used stratified sampling by sex, 10-year age groups, and 5–6 geographical Finnish areas.^13^ The Health 2000 Survey was conducted by the same institute. It comprised 8028 adults aged ≥30 years who were selected through (a regional) two-stage stratified cluster sampling to represent the Finnish adult population. Both surveys used consistent methods, measurements and protocol,^13^^,^^14^ comprising a total of 27,383 participants. Definitions of baseline variables are described in Supplemental Material, http://links.lww.com/HC9/B940. All participants provided signed informed consent. All surveys were approved by the Coordinating Ethical Committee of the Helsinki and Uusimaa Hospital District or the Institutional Review Board of the National Public Health Institute in Helsinki, Finland. In accordance with the Finnish Biobank Act, samples collected in both previous studies were transferred to the THL Biobank in 2015. Data from these surveys were linked to national registries using a unique personal identity code assigned to all Finnish residents. Follow-ups for deaths/hospitalizations were conducted until December 2019, and for malignancies until December 2021.

### UK Biobank dataset used for longitudinal analyses

The UK Biobank is a community cohort study of more than half a million individuals in the United Kingdom. Participants were interviewed between 2006 and 2010 at 22 assessment centers across England, Scotland, and Wales. All individuals aged 40–69 years and living within 25 miles of an assessment center (≈9 million persons) were invited to participate. Participants completed a comprehensive health questionnaire, underwent a physical examination, and donated biological specimens. Follow-up data are supplied through record linkage with mortality, hospital admission, and cancer registries in the United Kingdom,^15^ with censoring on November 30, 2022. The UK Biobank study was approved by the UK North West Multicentre Research Ethics Committee. Informed consent was obtained from each participant, with some withdrawing it later.

### US National Health and Nutrition Examination Survey used for cross-sectional analyses

The National Health and Nutrition Examination Survey (NHANES) is a cross-sectional, nationally representative survey conducted by the National Center for Health Statistics (NCHS), which collects (data regarding various) health and nutritional information of the US population. The survey employs a complex, multistage sampling design to ensure representativeness. Here, we used the NHANES 2017–2020 sample with available data regarding LSMs. The NHANES was approved by the NCHS Ethics Review Board, with no additional ethics approval being required for specific studies based on NHANES data. LSMs and the controlled attenuation parameter (CAP) using the FibroScan model 502 V2 Touch were measured for all participants aged ≥12 years. The exclusion criteria were inability to lie on the exam table, pregnancy, implanted electronic medical device, having a bandage, or lesions on the right abdomen by the ribs.

### Study exclusions

In the Finnish and UK Biobank datasets, we excluded participants with missing registry data, known baseline liver disease, chronic viral hepatitis, age <40 or >70 years at baseline, or missing data on crucial risk factors (Supplemental Figure S1, http://links.lww.com/HC9/B940). In the NHANES dataset, we excluded individuals without complete LSMs, which were defined as having a fasting time ≥3 hours before LSM and ≥10 stiffness measurements with an interquartile range of <30% from the median.

### Risk factors

Based on guidelines from major hepatology societies and findings from community-based screening studies,^1^^–^^8^^,^^16^ we identified established risk factors for liver fibrosis screening that are consistently emphasized for use in clinical practice: alcohol use, body mass index (BMI), diabetes, metabolic risk factors, and elevated LFTs. These factors are recommended for identifying individuals at risk of advanced fibrosis in the general population and primary care settings.^1^^,^^2^ The rationale was to capture individuals with any increased risk broadly, aiming to identify most of those who would go on to develop clinical LREs. We excluded studies focusing solely on patients with an established diagnosis of liver disease to align our approach with population-level screening strategies.

We applied several previously reported cutoffs for alcohol use and BMI (Supplemental Table S1, http://links.lww.com/HC9/B940).^5^^–^^8^ Regarding elevated LFTs, we considered ALT levels >35 U/L and >45 U/L for women and men, respectively, with or without a concomitant AST-to-ALT ratio >0.8.^7^ We considered the number of metabolic risk factors (0–5) present as per the steatotic liver disease nomenclature criteria.^17^ Moreover, we evaluated the non-laboratory version of the Chronic Liver Disease (CLivD) risk prediction score, which has been validated for screening individuals at high risk of LREs^10^^,^^18^^–^^21^ and considers age, sex, alcohol use (grams/week), diabetes, waist–hip ratio, and smoking status in its calculations. We used previously established cutoffs for the CLivD score (minimal, low, intermediate, and high).^10^ Taken together, we considered the following risk factors: alcohol use (4 cutoffs), BMI (3 cutoffs: 25, 30, and 35 kg/m^2^), number of metabolic risk factors (1, 2, or ≥3), diabetes, elevated LFTs (2 different definitions), and the CLivD score (3 different cutoffs). Supplemental Table S1, http://links.lww.com/HC9/B940, details the definitions and cutoffs. All possible combinations of risk factors and cutoffs yielded 1919 unique combinations (Supplemental Material, http://links.lww.com/HC9/B940), with at-risk individuals being identified by the presence of any of the risk factors.

### Outcomes

The primary liver-related outcome was hospitalization, cancer, or death related to liver cirrhosis, that is, a composite endpoint defined by the International Classification of Diseases 10th revision codes specified in Supplemental Table S2, http://links.lww.com/HC9/B940, as described in a recent consensus statement.^22^ In the NHANES dataset, the primary outcome was an LSM ≥12 kPa, which is a commonly used cutoff for ruling in compensated advanced chronic liver disease.^1^^,^^2^^,^^23^ We considered an LSM ≥15 kPa as a secondary outcome.

### Performance methods for LREs

We evaluated the time-dependent predictive ability of each risk factor combination for the 10-year risk of LREs in the Finnish and UK Biobank datasets, separately. Here, we set a 90% minimum sensitivity level as our primary benchmark. Risk factor combinations meeting the minimum sensitivity level were ranked according to their specificity, with the resulting top 5 combinations being selected. Further analyses at varying sensitivity thresholds (50%–90% in 5% increments) were performed to ensure robustness and elucidate the balance between sensitivity and specificity.

Since socioeconomic disparities may affect liver diseases^24^ and the UK Biobank cohort is not representative of the UK general population in this respect,^25^ we studied the performance of the risk factor combinations in quartiles based on the Townsend deprivation index, with the first (lowest index) and last (highest index) quartiles representing the least and most deprived groups, respectively.

In the UK Biobank cohort with available data to calculate the FIB-4 index, we assessed the predictive performance of a sequential strategy for the 10-year risk of LREs, where individuals with one or more risk factors are referred for FIB-4 testing. Here, a positive case was defined as being at risk and having FIB-4 ≥1.3, and a negative case as either having no risk factors or FIB-4 <1.3.

### Validation against LSM

In the US-based NHANES dataset, we validated the top 5 risk factor combinations of each of the other 2 datasets for their discriminatory performance in detecting an LSM ≥12 kPa. Further, we analyzed the performance of the sequential screening process, where a positive case was defined as being at risk and having FIB-4 ≥1.3, in detecting an LSM ≥12 kPa. LSM ≥15 kPa was considered in sensitivity analyses. Finally, we evaluated the performance of a screening strategy based on an approach that refers individuals for fibrosis evaluation if they present with elevated ALT levels or liver steatosis, as indicated by a Fibroscan-measured CAP value ≥288 dB/m.

### Statistical analyses

Group comparisons were performed using the chi-squared or *t* tests, as appropriate. Performance measures included sensitivity, specificity, and positive and negative predictive values. Additionally, we calculated the true and false positives and negatives, as well as the false positive fraction, that is, the population fraction considered at-risk but lacking the outcome. Time-dependent performance measures were calculated using the timeROC package in R. We estimated the cumulative probability of the LREs using the nonparametric cumulative incidence function (Aalen-Johansen), where death without an LRE was considered a competing-risk event. Statistical significance was set at a two-tailed *p* value <0.05. Statistical analyses were performed using R software, version 4.2.2.

## RESULTS

The Finnish, UK Biobank, and US-based NHANES datasets comprised 15,057, 454,990, and 3367 individuals, respectively. The distributions of age and sex were similar among these cohorts, while diabetes and metabolic risk factors were most common in the NHANES cohort, followed by the Finnish cohort (Table 1). The NHANES cohort had the highest mean BMI and lowest proportion of risky alcohol users than the other cohorts. In the Finnish cohort, there were 110 incident LREs and 1751 non-liver deaths during a median follow-up of 12.0 years (interquartile range [IQR]: 8.0–17.0). In the UK Biobank cohort, there were 2972 incident LREs and 36,717 non-liver deaths during a median follow-up of 13.7 years (IQR: 13.0–14.5). In the NHANES sample, there were 119 (3.5%) and 72 (2.1%) cases with LSMs ≥12 kPa and ≥15 kPa, respectively.

**TABLE 1:** Baseline demographics in the Finnish, UK Biobank, and US datasets

Description	Finnish cohorts	UK Biobank cohort	NHANES sample
Participants	15,057	454,990	3367
Age	54.6 (8.6)	56.5 (8.1)	55.4 (8.7)
Female, n (%)	7946 (52.8)	246,933 (54.3)	1714 (50.9)
Body mass index, kg/m^2^	27.4 (4.7)	27.4 (4.8)	30.4 (6.9)
Diabetes, n (%)	1457 (9.7)	26,130 (5.7)	776 (23.0)
Number of metabolic syndrome components, n (%)			
0	1315 (8.7)	88,710 (19.5)	158 (4.7)
1	3190 (21.2)	134,605 (29.6)	556 (16.5)
2	4665 (31.0)	121,926 (26.8)	955 (28.4)
3	3601 (23.9)	81,871 (18.0)	946 (28.1)
4	1720 (11.4)	24,217 (5.3)	551 (16.4)
5	566 (3.8)	3661 (0.8)	201 (6.0)
Alcohol use (grams of ethanol per week), n (%)			
≥168 g/wk for men or ≥112 g/wk for women	2566 (17.0)	116,205 (25.5)	223 (6.6)
≥210 g/wk for men or ≥140 g/wk for women	1885 (12.5)	86,387 (19.0)	172 (5.1)
≥400 g/wk for men or ≥280 g/wk for women	626 (4.2)	17,054 (3.7)	62 (1.8)
≥420 g/wk for men or ≥350 g/wk for women	518 (3.4)	11,808 (2.6)	46 (1.4)
Liver function tests, n (%)			
ALT >35 U/L for women and >45 U/L for men	1881 (12.5)	34,851 (7.7)	267 (7.9)
AST:ALT ratio >0.8 when ALT >35 U/L for women and >45 U/L for men	641 (4.3)	13,361 (2.9)	74 (2.2)
CLivD_non-lab_, _n (%)_			
Minimal	5244 (34.8)	147,243 (32.4)	1201 (35.7)
Low	8835 (58.7)	288,995 (63.5)	2023 (60.4)
Intermediate	671 (4.5)	14,702 (3.2)	95 (2.8)
High	307 (2.0)	4050 (0.9)	39 (1.2)
FIB-4, n (%)	n.a.		
<1.30		292,213 (65.9)	2413 (71.7)
≥1.30		151,311 (34.1)	954 (28.3)
Liver steatosis (CAP ≥288 dB/m), n (%)	n.a.	n.a.	1404 (41.7)
LSM ≥12 kPa, n (%)	n.a.	n.a.	119 (3.5)
LSM ≥15 kPa, n (%)	n.a.	n.a.	72 (2.1)

Abbreviations: CAP, controlled attenuation parameter; CLivD, Chronic Liver Disease risk score; FIB-4, fibrosis-4; LSM, liver stiffness measurement; n.a., not available; NHANES, National Health and Nutrition Examination Survey.

### Performance of risk factors in the Finnish dataset

Table 2 displays the top 5 risk factor combinations in the Finnish dataset with the highest specificity and ≥90% sensitivity for the 10-year risk of LREs. All these strategies incorporated the CLivD score, hazardous alcohol use, 3 or more metabolic risk factors, and elevated LFTs. Two strategies additionally included a BMI ≥35 kg/m^2^, and one also considered the presence of diabetes. The optimal cutoff CLivD score was set at intermediate or high risk relative to minimal or low risk. The optimal cutoff values for alcohol use for the purpose of liver disease screening were ≥210 g/week for men and ≥140 g/week for women. The specificities for these strategies ranged from 49.5% to 51.9%, with all exhibiting a sensitivity of 91.4%. Supplemental Table S3, http://links.lww.com/HC9/B941, shows the results for all 1919 combinations.

**TABLE 2:** Performance measures for predicting the 10-year risk of liver-related events of the 5 risk factor strategies with the highest specificity in the Finnish dataset within a minimum sensitivity level of 90%, 85%, or 80%

	Components and cutoffs of the risk factor strategy										
	Alcohol use (g/wk in men/women)	Number of metabolic syndrome components	Body mass index (kg/m^2^)	Diabetes	ALT >35 U/L for women and >45 U/L for men	AST:ALT ratio >0.8 when ALT >35 U/L for women and >45 U/L for men	CLivD score	Sensitivity (%)	Specificity (%)	PPV (%)	NPV (%)
Sensitivity ≥90%											
Strategy 1	≥210/140	≥3	—	—	—	Yes	Intermediate–high	91.4	51.9	0.8	95.5
Strategy 2	≥210/140	≥3	—	Yes	—	Yes	Intermediate–high	91.4	50.9	0.8	95.6
Strategy 3	≥210/140	≥3	≥35	—	—	Yes	Intermediate–high	91.4	50.7	0.8	95.5
Strategy 4	≥210/140	≥3	≥35	Yes	—	Yes	Intermediate–high	91.4	49.7	0.8	95.6
Strategy 5	≥210/140	≥3	—	—	Yes	—	Intermediate–high	91.4	49.5	0.8	95.4
Sensitivity ≥85%											
Strategy 1	≥168/112	—	≥30	—	Yes	—	—	86.4	58.5	0.9	94.5
Strategy 2	≥168/112	—	≥30	—	Yes	—	High	86.4	58.5	0.9	94.5
Strategy 3	≥168/112	—	≥30	—	Yes	—	Intermediate–high	86.4	58.3	0.9	94.7
Strategy 4	≥420/350	≥3	—	—	—	Yes	—	86.0	56.8	0.8	95.3
Strategy 5	≥420/350	≥3	—	—	—	Yes	High	86.0	56.7	0.8	95.4
Sensitivity ≥80%											
Strategy 1	—	—	≥30	—	Yes	—	Intermediate–high	80.9	66.7	1.0	94.9
Strategy 2	≥420/350	—	≥30	—	Yes	—	Intermediate–high	80.9	66.2	1.0	94.9
Strategy 3	≥400/280	—	≥30	—	Yes	—	Intermediate–high	80.9	65.9	1.0	94.9
Strategy 4	—	—	≥30	Yes	Yes	—	Intermediate–high	80.9	63.5	0.9	95.2
Strategy 5	≥420/350	—	≥30	Yes	Yes	—	Intermediate–high	80.9	63.1	0.9	95.2

Abbreviations: CLivD, Chronic Liver Disease risk score; NPV, negative predictive value; PPV, positive predictive value.

Adjusting the minimum sensitivity to 85% or 80% introduced greater variation in the top 5 combinations, especially in the alcohol use cutoffs (Table 2). When BMI was included, the threshold dropped to ≥30 kg/m^2^. All strategies at these sensitivities included elevated LFTs, with most incorporating the CLivD score. The specificities were 56.7%–58.5% and 63.1%–66.7% at 85% and 80% minimum sensitivity levels, respectively.

### Performance of risk factor combinations in the UK Biobank population

In the UK Biobank dataset, all strategies with a 90% minimum sensitivity level incorporated the CLivD score, at least 3 metabolic risk factors, and elevated LFTs (Table 3). Moreover, 3 accounted for alcohol use with varying thresholds, and 1 considered the presence of diabetes. Notably, the CLivD score threshold was set to a broader low–high-risk category, which was lower than that suggested for the Finnish cohort. The specificities for the most effective strategies (27.4%–27.6%) were lower than those for the Finnish dataset. Considering socioeconomic disparities, both sensitivity and specificity were marginally higher in the most deprived quartile than in the least deprived quartile for the top 5 strategies (Supplemental Table S4, http://links.lww.com/HC9/B940). Supplemental Tables S5–S7, http://links.lww.com/HC9/B941, shows the results for all 1919 combinations.

**TABLE 3:** Performance measures for predicting the 10-year risk of liver-related events of the 5 risk factor strategies with the highest specificity in the UK Biobank dataset within a minimum sensitivity level of 90%

	Individual entry criteria and their cutoffs for each strategy										
Sensitivity ≥90%	Alcohol use (g/wk in men/women)	Number of metabolic syndrome components	Body mass index (kg/m^2^)	Diabetes	ALT >35 U/L for women and >45 U/L for men	AST:ALT ratio >0.8 when ALT >35 U/L for women and >45 U/L for men	CLivD score	Sensitivity (%)	Specificity (%)	PPV (%)	NPV (%)
Strategy 1	—	≥3	—	—	—	Yes	Low–high	90.6	27.6	0.5	97.3
Strategy 2	≥400/280	≥3	—	—	—	Yes	Low–high	90.6	27.6	0.5	97.3
Strategy 3	≥420/350	≥3	—	—	—	Yes	Low–high	90.6	27.6	0.5	97.3
Strategy 4	≥210/140	≥3	—	—	—	Yes	Low–high	90.6	27.5	0.5	97.3
Strategy 5	—	≥3	—	Yes	—	Yes	Low–high	90.7	27.4	0.5	97.3

Abbreviations: CLivD, Chronic Liver Disease risk score; NPV, negative predictive value; PPV, positive predictive value.

Evaluation of the sequential screening strategy where a positive case is defined as being at risk and having an FIB-4 ≥1.3 revealed that the sensitivities and specificities fell to 68% and 67%, respectively, for each of the top 5 combinations.

### The impact of varying minimum sensitivity levels

**FIGURE 1:**
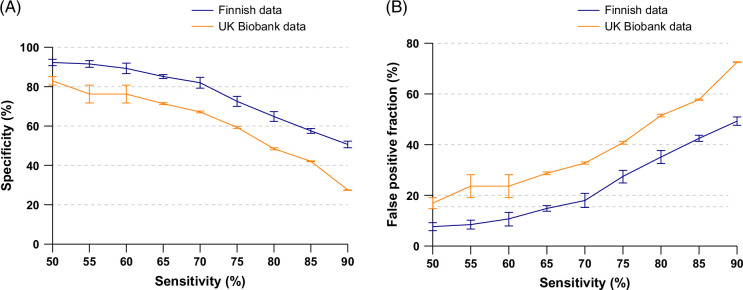
Performances of top risk factor combinations. Variations in (A) specificity and (B) false positive fraction among the top 5 risk factor combinations across a range of predefined minimum sensitivity levels (≥50% to ≥90%, in 5% increments) in the Finnish and UK Biobank datasets. The top 5 risk factor combinations were selected based on the specificity level with a particular minimum sensitivity level. The outcome was liver-related events within 10 years. False positive fraction refers to the proportion of the entire population that is considered at-risk but does not have a liver-related event within 10 years.

Next, we adjusted the minimum sensitivity thresholds from 50% to 90% in 5% increments, selecting the 5 most specific combinations at each sensitivity level. Figure 1 shows that at a 50% sensitivity threshold, there were >90% and >80% specificities for these top strategies in the Finnish and UK Biobank datasets, respectively. These specificity estimates decreased with higher minimum sensitivity requirements. Across all minimum sensitivity levels, specificities remained consistently higher in the most deprived quartile than in the least deprived quartile in the UK Biobank cohort (Supplemental Figure S2, http://links.lww.com/HC9/B940). The associations between false positive fractions and the minimum sensitivity requirement are shown in Figure 1.

### Validation in the NHANES sample

Among the top 5 combinations in the Finnish dataset, 2 showed a >90% sensitivity for detecting an LSM ≥12 kPa in the NHANES sample, with specificities of 38% and 41% (Table 4). Both combinations were based on the CLivD score, hazardous alcohol use, BMI ≥35 kg/m^2^, 3 or more metabolic risk factors (=metabolic syndrome), and/or the AST-to-ALT ratio; further, 1 of them was additionally based on the presence of diabetes (Table 4). Both combinations also yielded >90% sensitivities for detecting an LSM ≥15 kPa.

**TABLE 4:** External validation of the 5 best-performing risk factor strategies from the Finnish and UK Biobank datasets in the US population-based dataset (NHANES) for predicting liver stiffness measurement (LSM) ≥12 kPa or ≥15 kPa

	Individual entry criteria and their cutoffs for each strategy										
	Alcohol use (g/wk in men/women)	Number of metabolic syndrome components	Body mass index (kg/m^2^)	Diabetes	ALT >35 U/L for women and >45 U/L for men	AST:ALT ratio >0.8 when ALT >35 U/L for women and >45 U/L for men	CLivD score	Sensitivity (%)	Specificity (%)	PPV (%)	NPV (%)
LSM ≥12 kPa											
Strategy 1 (Finnish)	≥210/140	≥3	—	—	—	Yes	Intermediate–high	79.0	46.8	5.2	98.4
Strategy 2 (Finnish)	≥210/140	≥3	—	Yes	—	Yes	Intermediate-high	82.4	43.6	5.1	98.5
Strategy 3 (Finnish)	≥210/140	≥3	≥35	—	—	Yes	Intermediate–high	90.8	40.9	5.3	99.2
Strategy 4 (Finnish)	≥210/140	≥3	≥35	Yes	—	Yes	Intermediate–high	90.8	38.1	5.1	99.1
Strategy 5 (Finnish)	≥210/140	≥3	—	—	Yes	—	Intermediate–high	79.8	45.1	5.1	98.4
Strategy 1 (UK)	—	≥3	—	—	—	Yes	Low–high	94.1	24.3	4.4	99.1
Strategy 2 (UK)	≥400/280	≥3	—	—	—	Yes	Low–high	94.1	24.3	4.4	99.1
Strategy 3 (UK)	≥420/350	≥3	—	—	—	Yes	Low–high	94.1	24.3	4.4	99.1
Strategy 4 (UK)	≥210/140	≥3	—	—	—	Yes	Low–high	94.1	24.3	4.4	99.1
Strategy 5 (UK)	—	≥3	—	Yes	—	Yes	Low–high	95.0	24.0	4.4	99.2
LSM ≥15 kPa											
Strategy 1 (Finnish)	≥210/140	≥3	—	—	—	Yes	Intermediate–high	80.6	46.5	3.2	99.1
Strategy 2 (Finnish)	≥210/140	≥3	—	Yes	—	Yes	Intermediate–high	86.1	43.3	3.2	99.3
Strategy 3 (Finnish)	≥210/140	≥3	≥35	—	—	Yes	Intermediate–high	90.3	40.4	3.2	99.5
Strategy 4 (Finnish)	≥210/140	≥3	≥35	Yes	—	Yes	Intermediate–high	90.3	37.6	3.1	99.4
Strategy 5 (Finnish)	≥210/140	≥3	—	—	Yes	—	Intermediate–high	80.6	44.7	3.1	99.1
Strategy 1 (UK)	—	≥3	—	—	—	Yes	Low–high	94.4	24.0	2.6	99.5
Strategy 2 (UK)	≥400/280	≥3	—	—	—	Yes	Low–high	94.4	24.0	2.6	99.5
Strategy 3 (UK)	≥420/350	≥3	—	—	—	Yes	Low–high	94.4	24.0	2.6	99.5
Strategy 4 (UK)	≥210/140	≥3	—	—	—	Yes	Low–high	94.4	24.0	2.6	99.5
Strategy 5 (UK)	—	≥3	—	Yes	—	Yes	Low–high	95.8	23.8	2.7	99.6

Abbreviations: CLivD, Chronic Liver Disease risk score; NHANES, National Health and Nutrition Examination Survey; NPV, negative predictive value; PPV, positive predictive value.

All top 5 combinations in the UK Biobank dataset showed >90% sensitivity and 24% specificity for detecting an LSM ≥12 kPa in the NHANES sample (Table 4), with similar findings for detecting an LSM ≥15 kPa.

**FIGURE 2:**
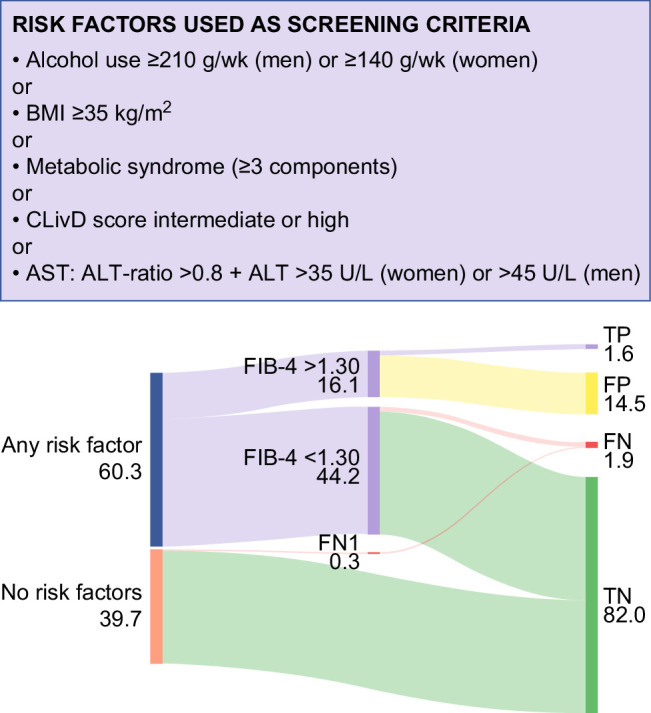
Sankey diagram for sequential liver stiffness screening. Participants from the NHANES sample are first categorized according to the presence of risk factors. At-risk individuals were further assessed using FIB-4 (cutoff: 1.3) for LSM ≥12 kPa detection. The outcomes are summarized in the right panel, showing true positives, false positives, false negatives, and true negatives. FN1 refers to those classified as false negatives without FIB-4 measurements. All numbers shown are percentages of the entire sample. Abbreviations: BMI, body mass index; CLivD score, Chronic Liver Disease risk score; FIB-4, fibrosis-4; FN, false negatives; FP, false positives; NHANES, National Center for Health Statistics; TN, true negatives; TP, true positives.

Evaluation of the sequential screening strategy where a positive case is defined as being at-risk (=having at least one of the risk factors) and having a FIB-4 ≥1.3 revealed a 45% sensitivity for detecting an LSM ≥12 kPa, with a specificity, positive predictive value, and negative predictive value of 85%, 10%, and 98%, respectively. Figure 2 shows the flow of individuals through this sequential strategy. Considering an LSM ≥15 kPa as the outcome, this strategy had 53% sensitivity and 85% specificity.

### Screening based on a steatosis diagnosis or elevated ALT in the US sample

In the detection of LSM ≥12 kPa, the approach relying on a CAP-based steatosis diagnosis had lower sensitivity (77% vs. 91%) but higher specificity (60% vs. 41%) than the risk factor-based strategy. The sequential strategy based on a steatosis diagnosis followed by an FIB-4 ≥1.3 showed 36% sensitivity and 91% specificity for detecting an LSM ≥12 kPa. The approach based on elevated ALT had very low sensitivity (24%) but high specificity (93%). The sequential strategy based on elevated ALT followed by an FIB-4 ≥1.3 showed 17% sensitivity and 97% specificity for detecting an LSM ≥12 kPa. Supplemental Figure S3, http://links.lww.com/HC9/B940 shows the flow of individuals through both aforementioned sequential strategies.

In additional exploratory analyses, we reduced the CAP cutoff to achieve ≥90% sensitivity for detecting LSM ≥12 kPa. With the CAP cutoff set at 228 dB/m, we achieved 91% sensitivity but 23% specificity.

### Validation of prognostic findings from the Finnish cohort in the UK Biobank cohort

**FIGURE 3:**
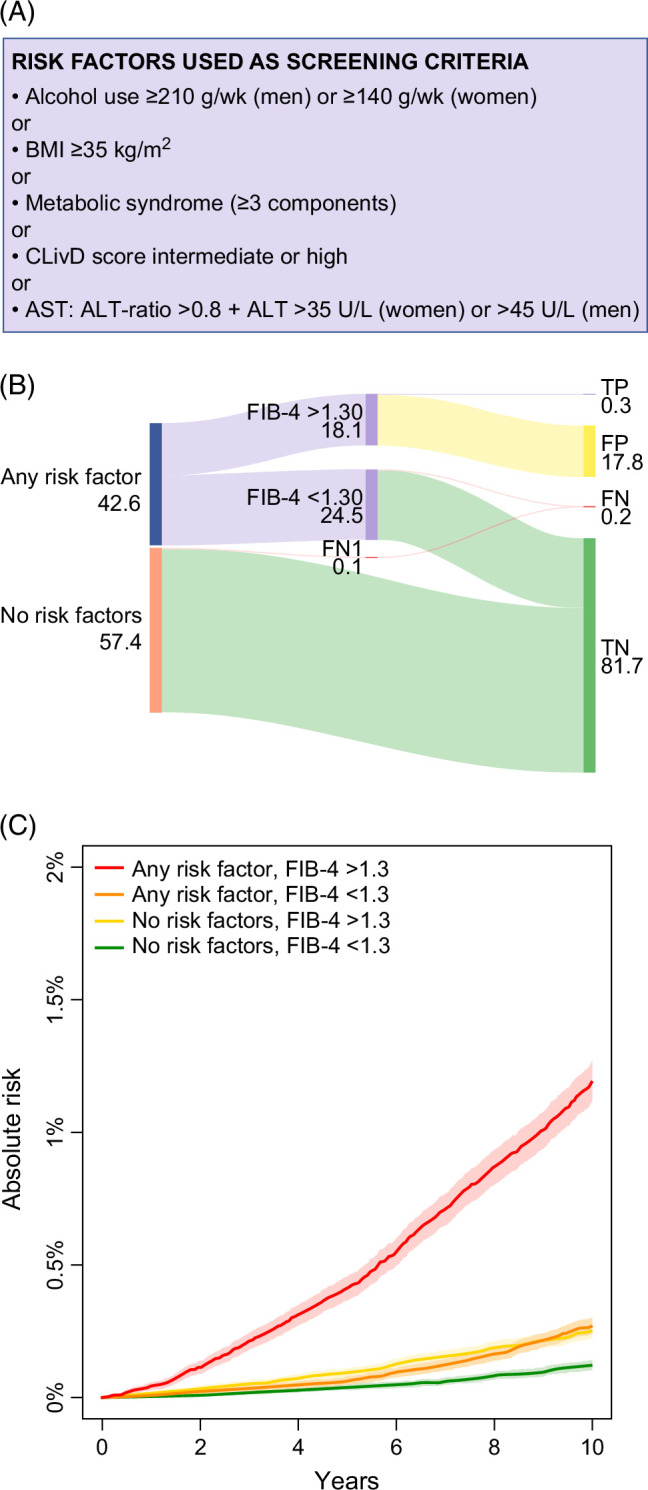
Sequential use of risk factors and FIB-4 in prediction of clinical liver-related events within 10 years—external validation in the UK Biobank cohort. (A) Shows the risk factors and their definitions. (B) Shows a Sankey diagram displaying percentages of the entire population of true positives, false positives, false negatives, and true negatives. FN1 refers to those classified as false negatives without FIB-4 measurements. All numbers shown are percentages of the entire sample. (C) Shows the cumulative incidence stratified by the presence of any risk factors and FIB-4 < or ≥1.3. Abbreviations: BMI, body mass index; CLivD score, Chronic Liver Disease risk score; FIB-4, fibrosis-4; FN, false negatives; FP, false positives; TN, true negatives; TP, true positives.

Based on the findings above, we selected the risk factor strategy that was among the top 5 combinations in the Finnish dataset and performed best in the US sample. This risk factor strategy was based on the presence of at least one of these: harmful alcohol use, severe obesity, 3 or more metabolic factors, CLivD score intermediate or high, and elevated AST:ALT ratio (Figure 2). Next, we externally validated this strategy in the UK Biobank cohort and analyzed its performance in a sequential approach with FIB-4 (cutoff 1.3) for predicting LREs within 10 years. Figure 3 shows the flow of individuals through this sequential strategy as well as the cumulative incidences of LREs according to the presence of risk factors and FIB-4 ≥1.3. The 10-year cumulative incidence of LREs was higher in individuals with any risk factor and an FIB-4 score ≥1.3 than in those with either any risk factor or FIB-4 ≥1.3 alone or neither criteria (Figure 3). The sensitivity and specificity of this sequential strategy were 56% and 82%, respectively. This represents only a marginal improvement in specificity compared to a single-step, risk factor-only approach adjusted to the same level of sensitivity, as shown in Figure 1.

### Excluding the CLivD score

To simplify the risk factor strategy and acknowledge potential concerns regarding the availability of some CLivD score components, we evaluated its performance without the CLivD score. Excluding the CLivD score had minimal to no impact on sensitivities and specificities for LRE prediction in the Finnish cohort (sensitivity 89.9%, specificity 50.8%), the UK Biobank cohort (sensitivity 73.0%, specificity 58.1%), and for LSM ≥12 kPa in the NHANES sample (sensitivity 90.7%, specificity 41.1%). Performance also remained stable for the sequential strategy (risk factors → FIB-4) with a sensitivity of 45.3% and specificity of 85.0%.

## DISCUSSION

This study investigated the risk factors that should be used as entry criteria for liver fibrosis screening, ensuring a predetermined minimum sensitivity level of the entry criteria. This approach addressed the question of who should undergo liver fibrosis screening using FIB-4 and other noninvasive fibrosis tests. Our study did not employ a multivariable risk factor model; instead, we developed a system directing individuals to liver fibrosis testing based on the presence of any single risk factor. By not combining multiple factors, this approach avoids issues like collinearity and focuses on identifying at-risk individuals through standalone criteria.

We focused on individuals aged 40–70 years and only considered established risk factors and cutoffs, which align with recommendations from major clinical guidelines and community-based screening studies.^1^^–^^8^ The primary outcome was incident clinical LREs, and we validated our findings cross-sectionally in the NHANES sample for elevated LSM. Our findings provide valuable insights into the expected sensitivities and specificities for population-level liver disease screening in at-risk individuals.

Based on the selected risk factor combinations for the Finnish dataset, the presence of any one of these risk factors could be a prompter in screening programs to further assess for advanced liver fibrosis by FIB-4 or similar tests: alcohol use >210 (men)/140 (women) g/week, at least 3 metabolic components, BMI ≥35 kg/m^2^, intermediate–high CLivD score, and AST:ALT ratio >0.8 with elevated ALT levels.

Although the UK Biobank cohort is not fully representative of the general population,^25^ findings obtained using the UK Biobank sample reinforced our findings. Specifically, the emergent risk factors in this cohort were similar to those in the Finnish cohort but at lower cutoffs when ordered by specificity. Moreover, the specificities were generally lower in the UK Biobank than in the Finnish cohort. An overrepresentation of healthy individuals in the UK Biobank may skew predictive values of risk factors and impair the generalizability of the findings.^25^ Therefore, we analyzed performance according to sociodemographic disparities, with the same combinations showing higher specificities in the most deprived quartile than in the least deprived quartiles.

In our best-performing strategy, depending on the population, ≈40%–60% of individuals aged 40–70 years would be considered at risk, and thus require fibrosis screening. This is comparable to a hypothetical strategy where all individuals with steatotic liver disease (prevalence ~38%) would be targeted for fibrosis screening as recommended in current guidelines.^2^ However, guidelines discourage direct screening for steatosis,^1^^,^^2^ which renders a substantial proportion of individuals affected by steatotic liver disease unaware of their condition. Although the proportion of individuals requiring fibrosis screening may appear high, it would be lower in reality since the risk factor profile is not universally known across the entire population. Restricting the proportion referred for screening can also be achieved by targeting specific age segments as applied in population-level colorectal cancer screening. Importantly, this broader approach to screening aims to maximize the impact of downstream interventions on reducing population-level morbidity and mortality from liver-related outcomes.

The ClivD score allows the detection of at-risk individuals based on the coexistence or accumulation of several weak risk factors rather than the presence of a single strong risk factor.^10^ This could minimize the likelihood of overlooking individuals whose risk arises from a combination of moderate-level factors, such as moderate alcohol use, slight abdominal overweight, smoking, and high age. The CLivD score was incorporated in almost all top-performing risk factor combinations, indicating the relevance of concomitant presence of, and interactions between alcohol use and metabolic conditions in liver disease. Nonetheless, excluding the CLivD score as an entry criterion did not result in a meaningful loss of discriminatory performance, suggesting that a simplified strategy based on more widely available risk factor data is sufficient for effective first-step risk stratification.

Diabetes was not consistently included in the top-performing risk factor combinations. However, diabetes is a component of the metabolic syndrome and is factored into the CLivD score. Therefore, accounting for metabolic syndrome components and the CLivD score may facilitate identifying patients with diabetes requiring liver disease screening. On the other hand, a simplified approach without either the CLivD score or diabetes maintained robust predictive performance across datasets.

The AST-to-ALT ratio was included in almost all best-performing risk factor combinations, which supports the concept of reflex testing, where laboratories automatically determine the AST level if the cutoff ALT level is exceeded.^26^ Although abnormal LFTs are markers of liver injury rather than risk factors by a strict definition, the widespread use of these LFTs in routine primary care supports the consideration of abnormal LFTs at the start of liver fibrosis screening.^4^

A hypothetical screening program based on a CAP-based diagnosis of liver steatosis or elevated ALT showed inferior sensitivity than the risk factor-based approach, especially when combined with FIB-4. The sensitivity of elevated ALT was particularly low (24% alone and 17% when combined with FIB-4), which confirms the poor utility of elevated ALT alone in population-level screening for significant chronic liver disease.^27^

It is important to consider the measurement simplicity and availability of risk factors when selecting them for disease screening programs. All components of the best-performing risk factor combinations are widely available and inexpensive and, therefore, may be considered as starting points for population-based liver disease screening. Since screening programs never have 100% attendance, that is, not all at-risk individuals invited will attend, we considered it crucial to aim for a high minimum sensitivity requirement for the program itself. Accordingly, we set a 90% minimum sensitivity level as our primary benchmark. However, since this requirement is arbitrary, we also analyzed performance metrics at lower sensitivity requirements, with the previously identified risk factors still showing robust findings.

Simple noninvasive tests, including FIB-4, for advanced liver fibrosis are not recommended in general populations due to low sensitivity related to the spectrum effect; rather, they should only be used for at-risk populations.^1^^,^^9^^,^^28^ We aimed to define such an at-risk population based on risk factors supported by guidelines.^1^^,^^2^ However, FIB-4 emerged as a weak link in the screening strategy, given the low sensitivity in the sequential strategy where FIB-4 was assessed in at-risk individuals. Compared with the risk factor strategy alone, the sequential strategy incorporating FIB-4 showed a reduced overall sensitivity from 91% to 45%. Accordingly, there is an urgent need for a more sensitive first-line fibrosis test with sufficient discriminatory ability. Newer fibrosis tests, such as LiverRisk,^29^ LiverPRO,^30^ and CORE,^31^ which are also based on widely accessible and inexpensive laboratory markers, might potentially replace FIB-4 in the future as the first-line test in population-based settings if further validation studies confirm superior diagnostic or prognostic accuracy. Their use should be explored in settings similar to the current study.

In the sequential risk factor and FIB-4 approach, there was a high proportion of false positives (15% of the entire population). Although the identification of actually at-risk individuals may have minimal harm, false positivity has more relevant harmfulness since the individual proceeds to undergo more specific liver testing (FIB-4 and LSM). This results in unnecessary cost and resource use as well as detrimental effects on the quality of life and anxiety in the individual.

A limitation of our study is the absence of a single dataset including all the following: risk factors, blood-based fibrosis tests, LSMs, and incident LREs. Although the study utilized large samples from Finland, the United Kingdom, and the United States, generalizability needs to be confirmed in other populations with different genetic, environmental, or healthcare access factors. Our findings also require further validation in ethnically diverse populations. Moreover, a large prospective study is warranted to determine the most optimal starting point for liver disease screening.

In conclusion, we identified a set of risk factors that can effectively serve as starting points in population-level liver disease screening. Our suggested risk factor combination achieved >90% sensitivity for predicting the 10-year risk of LREs as well as LSM >12 kPa and LSM >15 kPa.

## Supplementary Material

## References

[1] European Association for the Study of the Liver. EASL Clinical Practice Guidelines on non-invasive tests for evaluation of liver disease severity and prognosis—2021 update. J Hepatol. 2021;75:659–689. 34166721 10.1016/j.jhep.2021.05.025

[2] Rinella ME Neuschwander-Tetri BA Siddiqui MS Abdelmalek MF Caldwell S Barb D. AASLD Practice Guidance on the clinical assessment and management of nonalcoholic fatty liver disease. Hepatology. 2023;77:1797–1835. 36727674 10.1097/HEP.0000000000000323 PMC10735173

[3] Ginès P Castera L Lammert F Graupera I Serra-Burriel M Allen AM. Population screening for liver fibrosis: Toward early diagnosis and intervention for chronic liver diseases. Hepatology. 2022;75:219–228. 34537988 10.1002/hep.32163

[4] Newsome PN Cramb R Davison SM Dillon JF Foulerton M Godfrey EM. Guidelines on the management of abnormal liver blood tests. Gut. 2018;67:6–19. 29122851 10.1136/gutjnl-2017-314924 PMC5754852

[5] El-Gohary M Moore M Roderick P Watkins E Dash J Reinson T. Local care and treatment of liver disease (LOCATE)—A cluster-randomized feasibility study to discover, assess and manage early liver disease in primary care. PLoS One. 2018;13:e0208798. 30576330 10.1371/journal.pone.0208798 PMC6303066

[6] Harman DJ Ryder SD James MW Jelpke M Ottey DS Wilkes EA. Direct targeting of risk factors significantly increases the detection of liver cirrhosis in primary care: A cross-sectional diagnostic study utilising transient elastography. BMJ Open. 2015;5:e007516. 10.1136/bmjopen-2014-007516 PMC4420978 25941185

[7] Chalmers J Wilkes E Harris R Kent L Kinra S Aithal GP. The development and implementation of a commissioned pathway for the identification and stratification of liver disease in the community. Frontline Gastroenterol. 2020;11:86–92. 32066993 10.1136/flgastro-2019-101177 PMC7025872

[8] Kjaergaard M Lindvig KP Thorhauge KH Andersen P Hansen JK Kastrup N. Using the ELF test, FIB-4 and NAFLD fibrosis score to screen the population for liver disease. J Hepatol. 2023;79:277–286. 37088311 10.1016/j.jhep.2023.04.002

[9] Åberg F Jula A Färkkilä M Salomaa V Erlund I Männistö S. Comparison of various strategies to define the optimal target population for liver fibrosis screening: A population-based cohort study. United European. Gastroenterol J. 2022;10:1020–1028. 10.1002/ueg2.12323 PMC9731656 36318497

[10] Åberg F Luukkonen PK But A Salomaa V Britton A Petersen KM. Development and validation of a model to predict incident chronic liver disease in the general population: The CLivD score. J Hepatol. 2022;77:302–311. 35271949 10.1016/j.jhep.2022.02.021

[11] Abeysekera KW Shearer J Tavabie OD Dillon JF Rowe IA. #FGDebate: Should we focus on detecting patients at risk of liver disease in the community. Frontline Gastroenterol. 2023;14:343–345. 37409342 10.1136/flgastro-2022-102330 PMC11138178

[12] Hagström H Nasr P Ekstedt M Hammar U Stål P Hultcrantz R. Fibrosis stage but not NASH predicts mortality and time to development of severe liver disease in biopsy-proven NAFLD. J Hepatol. 2017;67:1265–1273. 28803953 10.1016/j.jhep.2017.07.027

[13] Borodulin K Tolonen H Jousilahti P Jula A Juolevi A Koskinen S. Cohort Profile: The National FINRISK Study. Int J Epidemiol. 2018;47:696–696i. 29165699 10.1093/ije/dyx239

[14] Aromaa A Koskinen S. Health and Functional Capacity in Finland: Baseline Results of the Health 2000 Health Examination Survey. Finland, Series B 12/2004 Helsinki: Publications of National Public Health Institute; 2004.

[15] Sudlow C Gallacher J Allen N Beral V Burton P Danesh J. UK biobank: An open access resource for identifying the causes of a wide range of complex diseases of middle and old age. PLoS Med. 2015;12:e1001779. 25826379 10.1371/journal.pmed.1001779 PMC4380465

[16] Caballería L Pera G Arteaga I Rodríguez L Alumà A Morillas RM. High prevalence of liver fibrosis among European adults with unknown liver disease: A population-based study. Clin Gastroenterol Hepatol. 2018;16:1138–1145.e5. 29452268 10.1016/j.cgh.2017.12.048

[17] Rinella ME Lazarus JV Ratziu V Francque SM Sanyal AJ Kanwal F. A multisociety Delphi consensus statement on new fatty liver disease nomenclature. J Hepatol. 2023;79:1542–1556. 37364790 10.1016/j.jhep.2023.06.003

[18] Åberg F Britton A Luukkonen PK. Changes over time in the Chronic Liver Disease risk score predict liver-related outcomes: Longitudinal analysis of the Whitehall II study. Scand J Gastroenterol. 2022;58:170–177. 35989617 10.1080/00365521.2022.2113130

[19] Pang Y Åberg F Chen Z Li L Kartsonaki C; China Kadoorie Biobank Collaborative Group. Predicting risk of chronic liver disease in Chinese adults: External validation of the CLivD score. J Hepatol. 2024;80:e264–e266. 38181826 10.1016/j.jhep.2023.12.022 PMC7617155

[20] Song J Jiang ZG. A good step toward low-cost prognostication of liver-related outcome awaits more validation. J Hepatol. 2022;77:887–889. 35460724 10.1016/j.jhep.2022.04.008

[21] Åberg F Asteljoki J Männistö V Luukkonen PK. Combined use of the CLivD score and FIB-4 for prediction of liver-related outcomes in the population. Hepatology. 2024;80:163–172. 38112489 10.1097/HEP.0000000000000707 PMC11191041

[22] Shearer JE Gonzalez JJ Min T Parker R Jones R Su GL. Systematic review: Development of a consensus code set to identify cirrhosis in electronic health records. Aliment Pharmacol Ther. 2022;55:645–657. 35166399 10.1111/apt.16806 PMC9302659

[23] Papatheodoridi M Hiriart JB Lupsor-Platon M Bronte F Boursier J Elshaarawy O. Refining the Baveno VI elastography criteria for the definition of compensated advanced chronic liver disease. J Hepatol. 2021;74:1109–1116. 33307138 10.1016/j.jhep.2020.11.050

[24] Karlsen TH Sheron N Zelber-Sagi S Carrieri P Dusheiko G Bugianesi E. The EASL-Lancet Liver Commission: Protecting the next generation of Europeans against liver disease complications and premature mortality. Lancet. 2022;399:61–116. 34863359 10.1016/S0140-6736(21)01701-3

[25] Fry A Littlejohns TJ Sudlow C Doherty N Adamska L Sprosen T. Comparison of sociodemographic and health-related characteristics of UK Biobank participants with those of the general population. Am J Epidemiol. 2017;186:1026–1034. 28641372 10.1093/aje/kwx246 PMC5860371

[26] Birindelli S Pasqualetti S Panteghini M. Offering aspartate aminotransferase as a reflex test: An easy but effective way to improve appropriateness of laboratory requests. Am J Clin Pathol. 2018;149:456–457. 29547948 10.1093/ajcp/aqy015

[27] Harris R Harman DJ Card TR Aithal GP Guha IN. Prevalence of clinically significant liver disease within the general population, as defined by non-invasive markers of liver fibrosis: A systematic review. Lancet Gastroenterol Hepatol. 2017;2:288–297. 28404158 10.1016/S2468-1253(16)30205-9

[28] Graupera I Thiele M Serra-Burriel M Caballeria L Roulot D Wong GL-H. Low accuracy of FIB-4 and NAFLD fibrosis scores for screening for liver fibrosis in the population. Clin Gastroenterol Hepatol. 2022;20:2567–2576. 34971806 10.1016/j.cgh.2021.12.034

[29] Serra-Burriel M Juanola A Serra-Burriel F Thiele M Graupera I Pose E. Development, validation, and prognostic evaluation of a risk score for long-term liver-related outcomes in the general population: A multicohort study. Lancet. 2023;402:988–996. 37572680 10.1016/S0140-6736(23)01174-1

[30] Lindvig KP. Development, validation, and prognostic evaluation of LiverPRO for the prediction of significant liver fibrosis in primary care: A prospective cohort study. Lancet Gastroenterol Hepatol. 2025;10:55–67. 39674225 10.1016/S2468-1253(24)00274-7

[31] Strandberg R Talbäck M Hammar N Hagström H. OS-057-YI CORE: A new risk score measuring GGT, AST, and ALT outperforms FIB-4 when predicting the risk of cirrhosis in a primary care setting. J Hepatol. 2024;80:S40.

