# Supplementary material for: Evidence-based criteria for identifying at-risk individuals requiring liver disease screening
Source: Hepatol Commun. 2025 Mar 21;9(4):e0679. doi: 10.1097/HC9.0000000000000679 (PMC11927647; doi:10.1097/HC9.0000000000000679)
Supplement: Supplementary file 1 [file hc9-9-e0679-s001.docx]

**Supplementary Appendix**

**Evidence-based criteria for identifying** **at-risk individuals requiring liver disease screening**

Åberg Fredrik, Männistö Ville, Asteljoki Juho, Salomaa Veikko, Jula Antti,

Lundqvist Annamari, Männistö Satu, Perola Markus, Luukkonen Panu K

**Table of contents**

[Supplementary Methods 3](#_Toc169267642)

[Supplementary Table 1. The variables and their definitions and cutoffs used in the risk factor combination strategies. 6](#_Toc169267643)

[Supplementary Table 2. International Classification of Diseases 10 (ICD-10) codes used to define the liver-related outcome. 9](#_Toc169267644)

[Supplementary Table 3. Performance metrics for all 1919 risk factor combinations in the Finnish dataset 10](#_Toc169267645)

[Supplementary Table 4. Performance measures in the most and least deprived quartiles (Townsend index) of the five risk factor strategies with the highest specificity in the overall UK Biobank population at a minimum 90% sensitivity level for predicting the 10-year risk of liver-related events 11](#_Toc169267646)

[Supplementary Table 5. Performance metrics for all 1919 risk factor combinations in the overall UK Biobank dataset 14](#_Toc169267647)

[Supplementary Table 6. Performance metrics for all 1919 risk factor combinations in the most deprived quartile (Townsend deprivation index) of the UK Biobank dataset 14](#_Toc169267648)

[Supplementary Table 7. Performance metrics for all 1919 risk factor combinations in the least deprived quartile (Townsend deprivation index) of thr UK Biobank dataset 14](#_Toc169267649)

[Supplementary Figure 1: Flow chart of inclusion and exclusion criteria in the (A) Finnish dataset, (B) UK Biobank dataset, and (C) the National Health and Nutrition Examination Survey (NHANES) dataset. 15](#_Toc169267650)

[Supplementary Figure 2: The variation in specificity among the top five risk factor combinations across a range of predefined minimum sensitivity levels (≥50% to ≥90%, in 5% increments) in the UK Biobank cohort with comparison according to socioeconomic disparities determined using the Townsend deprivation index (least vs. most deprived quartiles). 16](#_Toc169267651)

[Supplementary Figure 3. Sankey diagram for sequential liver stiffness screening. Participants from the US NHANES sample are first categorized according to the presence of CAP-based liver steatosis (panel A) or elevated ALT (panel B). At-risk individuals are further assessed using FIB-4, with a cutoff of 1.3 for LSM ≥12 kPa detection. 17](#_Toc169267652)

[References 19](#_Toc169267653)

# Supplementary Methods

*Definition of baseline variables*

In the Finnish dataset, respondents were asked to report their frequency of consumption of alcoholic beverages during the previous year and the average amount they consumed per week during the previous month. Average alcohol intake (100% ethanol in grams per week) was calculated based on these data. In the UK Biobank dataset, alcohol consumption was evaluated using a self-administered questionnaire. Specifically, participants were questioned about their drinking status and average weekly or monthly consumption in terms of the number of glasses of red wine, champagne, white wine, pints of beer or cider, spirits, or glasses of fortified wine and other alcoholic drinks. For each participant, we calculated the mean consumption of ethanol in grams per week as described by Tavaglione et al.^1^, assuming 2 units of pure alcohol in a pint of beer or cider; 1.5 units in a glass (125 mL) of red wine, champagne, white wine, fortified wine, and other alcoholic drinks; and 1 unit in a measure (25 mL) of spirit, where one unit contains 8 g of pure ethanol. In the US NHANES dataset, alcohol intake was evaluated based on a quantity-frequency questionnaire. Average alcohol intake (100% ethanol in grams per week) was calculated based on these data.

Body mass index was calculated from measured body weight and height in all samples. In the Finnish dataset, diabetes was defined either by a fasting serum glucose ≥7.0 mmol/L, taking diabetes medication, or a history of diabetes diagnosis. In the UK Biobank dataset, diabetes was defined based on a baseline self-reported diabetes diagnosis, a registry code for diabetes, taking diabetic medication, serum glucose level ≥11.0 mmol/L, or a glycated hemoglobin (HbA1c) level ≥48 mmol/mol. In the US NHANES dataset, diabetes was defined based on a fasting blood glucose ≥7.0 mmol/L, glycated hemoglobin (HbA1c) >6.5%, or a history of diabetes diagnosis.

Metabolic risk factors (0–5 factors) were defined according to recent SLD nomenclature criteria^2^ using a waist circumference >94 cm for male and >80 cm for female; fasting glucose ≥5.6 mmol/l or ≥7.8 mmol/l at 2 h in the glucose tolerance test or a diagnosis of type 2 diabetes; blood pressure >130/85 mmHg or taking antihypertensive medication; triglycerides > 1.7 mmol/l or taking lipid medication; or HDL cholesterol ≤1.0 mmol/l (male) or ≤1.3 mmol/l (female) or taking lipid medication.

ALT and AST measurements were analyzed from blood samples collected at the initial visit.

The non-laboratory CLivD_non-lab_ score was calculated based on age, sex, smoking status (current vs. previous/never), alcohol use (number of weekly drinks, where 1 drink = 10 grams of ethanol), waist-to-hip ratio, and diabetes (yes vs. no) by the previously published equation^3^:

The CLivD_non-lab_ equation in R software language

data = name of the R data

AGE = age in years

SEX = men = 1; women = 2

WHR = waist hip ratio

ALCOHOL = number of weekly drinks (1 drink = 10 g ethanol)

Set to = 50, if >50 drinks per week

DIABETES = yes = 1; no = 0

SMOKING = current smoker = 1; never/previous smoker = 2

*CLivD_non-lab_*

data$modelnonlab <- (-8.0940103 +0.044177151* data$AGE +0.48927753*( data$WHR*10) +0.19222894* data$ALCOHOL -0.00015029544*pmax(data$ALCOHOL-0.1,0)^3 -0.0021265611*pmax(data$ALCOHOL-1,0)^3 +0.0029832769*pmax(data$ALCOHOL-3,0)^3 -0.00068765143*pmax(data$ALCOHOL-9,0)^3 -1.8769011e-05*pmax(data$ALCOHOL-33,0)^3 +0.69669285*(data$DIABETES=="1") +0.75968055*(data$SMOKING=="1")+ 0.63248362*(data$SEX=="2") -0.59146649*(data$SMOKING=="1")*(data$SEX=="2"))

*Cutoff values for risk groups*

CLivD_non-lab_

Minimal (15-yr risk <0.5%): < -0.412

Low (15-yr risk 0.5-4%): -0.413–1.912

Intermediate (15-yr risk 5-9%): 1.913–2.632

High (15-yr risk ≥10%): ≥ 2.633

# Supplementary Table 1. The variables and their definitions and cutoffs used in the risk factor combination strategies.

| Variable | Description | Cutoffs (Number) | Options (Cutoffs + None) | Categories | References |
| --- | --- | --- | --- | --- | --- |
| Alcohol use | Alcohol use categorization | 4 | 5 | Category 1: >168 g/week for men or >112 g/week for women  Category 2: ≥210 g/week for men or ≥140 g/week for women  Category 3: ≥400 g/week for men or ≥280 g/week for women  Category 4: ≥420 g/week for men or ≥350 g/week for women | [2,4–8] |
| BMI | Body Mass Index | 3 | 4 | 25, 30, or 35 kg/m² | [5,7,8] |
| METS | Number of metabolic risk factors | 3 | 4 | 1, 2, or ³3 metabolic risk factors | [2,5,8] |
| Diabetes | Diabetes | 1 | 2 | Presence or absence of diabetes | [5,8] |
| CLivD | Chronic Liver Disease risk score categorization | 3 | 4 | Category 1: Low or intermediate or high  Category 2: Intermediate or high  Category 3: High | [3] |
| LFTs | Alanine aminotransferase (ALT) and aspartate aminotransferase (AST) levels | 2 | 3 | 1: ALT >35 U/L for women, >45 U/L for men  2: AST:ALT ratio >0.8 when concomitantly ALT >35 U/L for women or >45 U/L for men | [4,6,7,9] |

**Calculation of the number of unique combinations:**

Total combinations = 5 (Alcohol use) × 4 (BMI) × 4 (METS) × 2 (Diabetes) × 4 (CLivD) × 3 (LFTs) – 1 = 1919

The equation includes one more option for each variable to account for a situation where the variable is not considered at all.

The "-1" at the end of the formula accounts for the exclusion of the scenario where no variables (risk factors) are selected.

# Supplementary Table 2. International Classification of Diseases 10 (ICD-10) codes used to define the liver-related outcome.

| **ICD‐10 code** | **Description** |
| --- | --- |
| K74.6 | Other and unspecified cirrhosis of the liver |
| K70.3 | Alcoholic cirrhosis of the liver |
| I85 | Oesophageal varices |
| I85.0 | With bleeding |
| I85.9 | Without bleeding |
| I98 | Oesophageal varices in diseases classified elsewhere |
| I98.2 | Without bleeding |
| I98.3 | With bleeding |
| K76.6 | Portal hypertension |
| K72.9 | Hepatic failure, unspecified |
| K76.7 | Hepatorenal syndrome |
| C22.0 | Hepatocellular carcinoma |

# Supplementary Table 3. Performance metrics for all 1919 risk factor combinations in the Finnish dataset

*Please see separate Excel file.*

# Supplementary Table 4. Performance measures in the most and least deprived quartiles (Townsend index) of the five risk factor strategies with the highest specificity in the overall UK Biobank population at a minimum 90% sensitivity level for predicting the 10-year risk of liver-related events

| **Sensitivity ≥90%** | **Alcohol use (grams/week in men/women)** | **Number of metabolic syndrome components** | **Body mass index (kg/m^2^)** | **Diabetes** | **ALT >35 U/L for women and >45 U/L for men** | **AST:ALT-ratio >0.8 when ALT >35 U/L for women and >45 U/L for men** | **CLivD score** | **Sensitivity** | **Specificity** | **PPV** | **NPV** |
| --- | --- | --- | --- | --- | --- | --- | --- | --- | --- | --- | --- |
| **Most deprived quartile** | | | | | | | | | | | |
| Strategy 1 | x | ≥3 | X | x | x | yes | low-high | 91.5 % | 30.0 % | 0.7 % | 96.8 % |
| Strategy 2 | ≥400/280 | ≥3 | X | x | x | yes | low-high | 91.5 % | 30.0 % | 0.7 % | 96.8 % |
| Strategy 3 | ≥420/350 | ≥3 | X | x | x | yes | low-high | 91.5 % | 30.0 % | 0.7 % | 96.8 % |
| Strategy 4 | ≥210/140 | ≥3 | X | x | x | yes | low-high | 91.5 % | 30.0 % | 0.7 % | 96.8 % |
| Strategy 5 | x | ≥3 | X | yes | x | yes | low-high | 91.5 % | 29.8 % | 0.7 % | 96.8 % |
| **Least deprived quartile** | | | | | | | | | | | |
| Strategy 1 | x | ≥3 | X | x | x | yes | low-high | 88.6 % | 26.1 % | 0.3 % | 97.6 % |
| Strategy 2 | ≥400/280 | ≥3 | X | x | x | yes | low-high | 88.6 % | 26.1 % | 0.3 % | 97.6 % |
| Strategy 3 | ≥420/350 | ≥3 | X | x | x | yes | low-high | 88.6 % | 26.1 % | 0.3 % | 97.6 % |
| Strategy 4 | ≥210/140 | ≥3 | X | x | x | yes | low-high | 88.6 % | 26.0 % | 0.3 % | 97.6 % |
| Strategy 5 | x | ≥3 | X | yes | x | yes | low-high | 88.9 % | 26.0 % | 0.3 % | 97.6 % |

# Supplementary Table 5. Performance metrics for all 1919 risk factor combinations in the overall UK Biobank dataset

Please see separate Excel file.

# Supplementary Table 6. Performance metrics for all 1919 risk factor combinations in the most deprived quartile (Townsend deprivation index) of the UK Biobank dataset

*Please see separate Excel file.*

# Supplementary Table 7. Performance metrics for all 1919 risk factor combinations in the least deprived quartile (Townsend deprivation index) of thr UK Biobank dataset

*Please see separate Excel file.*

# Supplementary Figure 1: Flow chart of inclusion and exclusion criteria in the (A) Finnish dataset, (B) UK Biobank dataset, and (C) the National Health and Nutrition Examination Survey (NHANES) dataset.





# Supplementary Figure 2: The variation in specificity among the top five risk factor combinations across a range of predefined minimum sensitivity levels (≥50% to ≥90%, in 5% increments) in the UK Biobank cohort with comparison according to socioeconomic disparities determined using the Townsend deprivation index (least vs. most deprived quartiles).

The top combinations were selected based on specificity under the requirement of a specific minimum sensitivity level. The outcome was liver-related events during a 10-year follow-up.


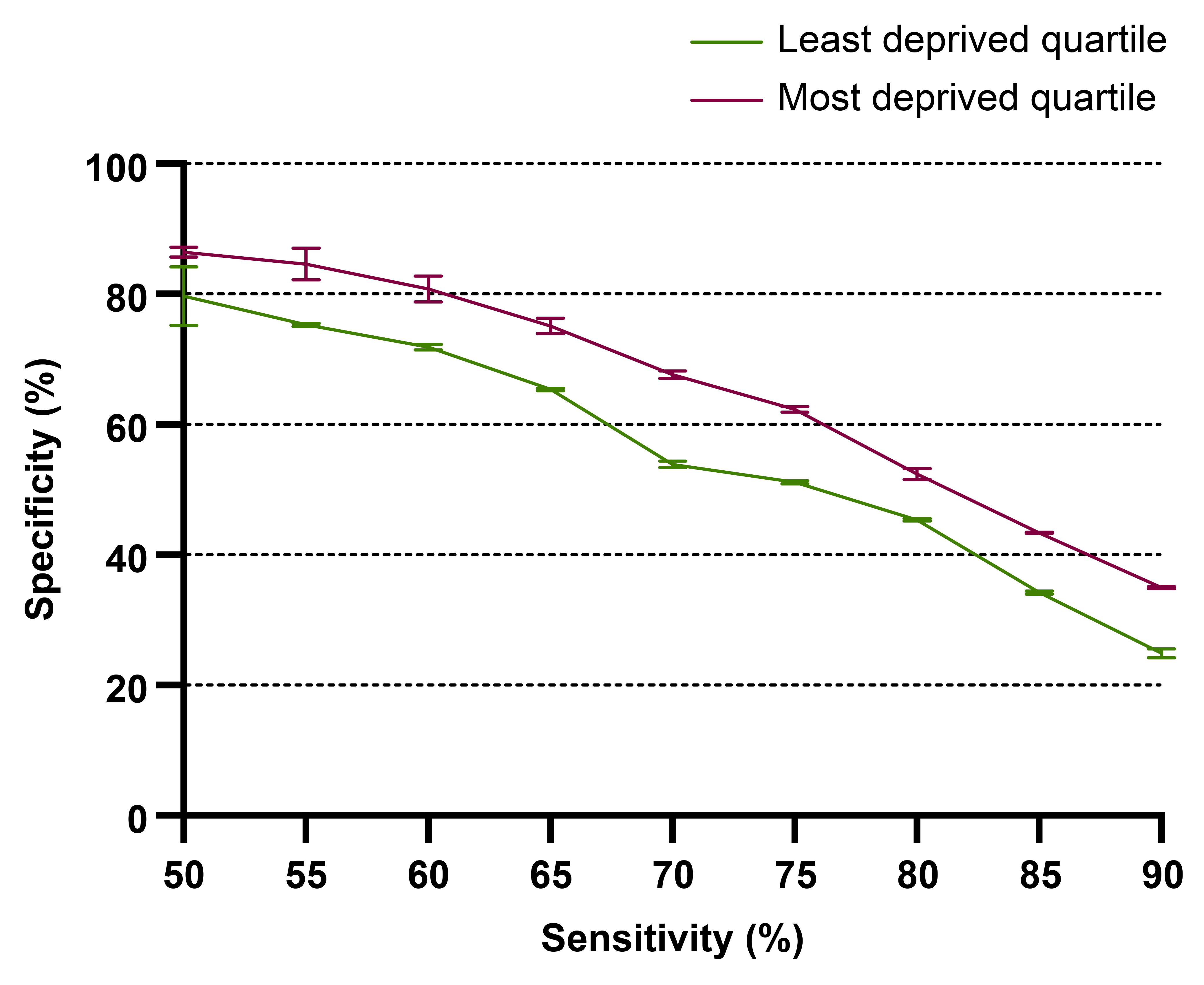


# Supplementary Figure 3. Sankey diagram for sequential liver stiffness screening. Participants from the US NHANES sample are first categorized according to the presence of CAP-based liver steatosis (panel A) or elevated ALT (panel B). At-risk individuals are further assessed using FIB-4, with a cutoff of 1.3 for LSM ≥12 kPa detection.

The outcomes are summarized in the right panel, showing true positives (TP), false positives (FP), false negatives (FN), and true negatives (TN). All numbers shown are percentages of the entire sample.


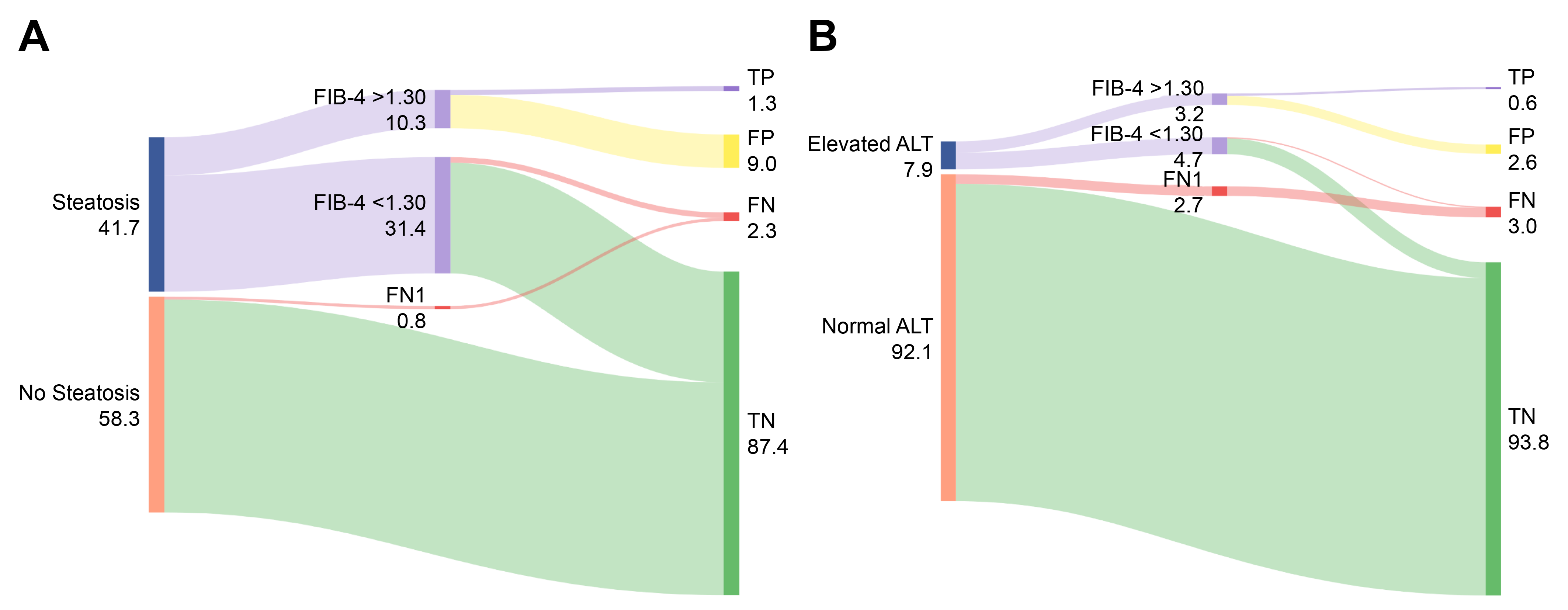


# References

[1] Tavaglione F, De Vincentis A, Jamialahmadi O, Pujia R, Spagnuolo R, Picardi A, et al. Inborn and acquired risk factors for severe liver disease in Europeans with type 2 diabetes from the UK Biobank. JHEP Rep 2021;3:100262. <https://doi.org/10.1016/j.jhepr.2021.100262>.

[2] Rinella ME, Lazarus JV, Ratziu V, Francque SM, Sanyal AJ, Kanwal F, et al. A multisociety Delphi consensus statement on new fatty liver disease nomenclature. J Hepatol 2023;79:1542–1556. <https://doi.org/10.1016/j.jhep.2023.06.003>.

[3] Åberg F, Luukkonen PK, But A, Salomaa V, Britton A, Petersen KM, et al. Development and validation of a model to predict incident chronic liver disease in the general population: the CLivD score. J Hepatol 2022;77:302–311. <https://doi.org/10.1016/j.jhep.2022.02.021>.

[4] Harman DJ, Ryder SD, James MW, Jelpke M, Ottey DS, Wilkes EA, et al. Direct targeting of risk factors significantly increases the detection of liver cirrho1sis in primary care: a cross-sectional diagnostic study utilising transient elastography. BMJ Open 2015;5:e007516. <https://doi.org/10.1136/bmjopen-2014-007516>.

[5] Rinella ME, Neuschwander-Tetri BA, Siddiqui MS, Abdelmalek MF, Caldwell S, Barb D, et al. AASLD Practice Guidance on the clinical assessment and management of nonalcoholic fatty liver disease. Hepatology 2023;77:1797–1835. [https://doi.org/10.1097/HEP.0000000000000323](https://doi.org/10.1097/hep.0000000000000323).

[6] El-Gohary M, Moore M, Roderick P, Watkins E, Dash J, Reinson T, et al. Local care and treatment of liver disease (LOCATE) – A cluster-randomized feasibility study to discover, assess and manage early liver disease in primary care. PLoS One 2018;13:e0208798. <https://doi.org/10.1371/journal.pone.0208798>.

[7] Chalmers J, Wilkes E, Harris R, Kent L, Kinra S, Aithal GP, et al. The development and implementation of a commissioned pathway for the identification and stratification of liver disease in the community. Frontline Gastroenterol 2020;11:86–92. <https://doi.org/10.1136/flgastro-2019-101177>.

[8] Kjaergaard M, Lindvig KP, Thorhauge KH, Andersen P, Hansen JK, Kastrup N, et al. Using the ELF test, FIB-4 and NAFLD fibrosis score to screen the population for liver disease. J Hepatol 2023;79:277–286. <https://doi.org/10.1016/j.jhep.2023.04.002>.

[9] Newsome PN, Cramb R, Davison SM, Dillon JF, Foulerton M, Godfrey EM, et al. Guidelines on the management of abnormal liver blood tests. Gut 2018;67:6–19. <https://doi.org/10.1136/gutjnl-2017-314924>.
